# Novel catalytically active Pd/Ru bimetallic nanoparticles synthesized by *Bacillus benzeovorans*

**DOI:** 10.1038/s41598-019-40312-3

**Published:** 2019-03-18

**Authors:** Jacob B. Omajali, Jaime Gomez-Bolivar, Iryna P. Mikheenko, Surbhi Sharma, Bayonle Kayode, Bushra Al-Duri, Dipanjan Banerjee, Marc Walker, Mohamed L. Merroun, Lynne E. Macaskie

**Affiliations:** 1 School of Biosciences, University of Birmingham, Edgbaston, Birmingham, B15 2TT UK; 2 Department of Microbiology, Faculty of Sciences, University of Granada, Campus Fuentenueva, 18071 Granada, Spain; 3 School of Chemical Engineering, University of Birmingham, Edgbaston, Birmingham, B15 2TT UK; 4 Dutch-Belgian Beamline (DUBBLE), ESRF - The European Synchrotron, 38043 Grenoble, France; 5 Department of Physics University of Warwick, Coventry, CV4 7AL United Kingdom; 6 Present Address: Department of Chemistry, Faculty of Sciences, Thompson Rivers University, 805 TRU Way, V2C 0C8, Kamloops, British Columbia Canada

## Abstract

*Bacillus benzeovorans* assisted and supported growth of ruthenium (bio-Ru) and palladium/ruthenium (bio-Pd@Ru) core@shell nanoparticles (NPs) as bio-derived catalysts. Characterization of the bio-NPs using various electron microscopy techniques and high-angle annular dark field (HAADF) analysis confirmed two NP populations (1–2 nm and 5–8 nm), with core@shells in the latter. The Pd/Ru NP lattice fringes, 0.231 nm, corresponded to the (110) plane of RuO_2._ While surface characterization using X-ray photoelectron spectroscopy (XPS) showed the presence of Pd(0), Pd(II), Ru(III) and Ru(VI), X-ray absorption (XAS) studies of the bulk material confirmed the Pd speciation (Pd(0) and Pd(II)- corresponding to PdO), and identified Ru as Ru(III) and Ru(IV). The absence of Ru–Ru or Ru–Pd peaks indicated Ru only exists in oxide forms (RuO_2_ and RuOH), which are surface-localized. X ray diffraction (XRD) patterns did not identify Pd-Ru alloying. Preliminary catalytic studies explored the conversion of 5-hydroxymethyl furfural (5-HMF) to the fuel precursor 2,5-dimethyl furan (2,5-DMF). Both high-loading (9.7 wt.% Pd, 6 wt.% Ru) and low-loading (2.4 wt.% Pd, 2 wt.% Ru) bio-derived catalysts demonstrated high conversion efficiencies (~95%) and selectivity of ~63% (~20% better than bio-Ru NPs) and 58%, respectively. These materials show promising future scope as efficient low-cost biofuel catalysts.

## Introduction

The synthesis of bimetallic nanoparticles (NPs) has attracted major interest, given their ubiquitous catalytic applications and the unique properties ascribed to synergistic interactions between the two metallic components. Extensive reviews summarize a variety of preparation routes, shapes, catalytic and optical properties of various bimetallic nanostructures^1^. The suitability of living organisms, including bacterial cells, to template and form catalytic mono-metallic NPs is well known e.g.^2,3^, but microbially-assisted fabrication of structured bimetallic NPs is hardly investigated.

A paradigm study^4^ investigated microbially-assisted production of gold-palladium core@shell NPs (bio-Au@Pd). An effective catalyst in the partial oxidation of benzyl alcohol, bio-Au@Pd was formed by ‘seeding’ of biogenic Pd(0)-NPs and galvanic reduction of Au(III) and eventual outward migration of oxidized Pd species, followed by the reduction of the latter under H_2_ to form a Pd(0) shell^4^. Synthesis of bio-Au@Pd NPs has been reported using various Gram-negative bacteria such as *Cupriavidus necator*^5^, *Desulfovibrio desulfuricans*^6^ and *Escherichia coli*^4^. The paradigm synthesis uses biochemical activity (e.g. hydrogenases) to reduce Pd(II) to Pd(0) to form ‘seeds’ that are patterned onto the biomatrix. At the pH of Pd(II) exposure (pH 2–3) cell viability and hydrogenase activity are rapidly lost; further catalyst synthesis is autocatalytic with reduction of the second metal under H_2_ via the Pd(0) ‘seeds’, resulting in the bimetallic^4^. This comprises chemical nanoparticles which retain the advantage of bio-scaffolding imprinted in the initial Pd-seeding step.

More recently, the identification of intracellular Au@Pd core@shell NPs (Supplementary Information Fig. S1) (as opposed to earlier reports of NP growth on bacterial cell surfaces) following deposition of Pd-NPs in the cytoplasm^7^ implies cellular uptake and trafficking mechanism(s) for metals with no known biological roles. Flow cytometry studies have established metabolic activity following uptake of Pd(II) and its intracellular reduction to Pd(0)^8^. Intracellular localization of Pd NPs was also reported for the Gram-positive organism *Bacillus benzeovorans*, with co-localization of cytoplasmic Pd with phosphate groups (Supplementary Information Fig. S2) but attempts to fabricate bimetallic NPs on these Pd-‘seeds’ are not reported using Ru; the chemical formation of bimetallic Pd@Ru NPs is under-examined, while their facile biofabrication remains unexplored.

Although fabrication of Pd@Ru NPs is rare, they have significant applications in ‘green’ chemistry where the two metals in this bimetallic or core@shell configuration are known to assist in enhancing the catalytic performance of the other. Recent research has highlighted the potential of modulating fcc and hcp structures of Ru to tailor the selectivity of bimetallic structures^9^. Some of the applications of Pd-Ru systems include: (1) hydrogenation of hex-1-ene to *n*-hexane (Pd_6_Ru_6_ clusters are several orders of magnitude more active than Pd alone^10^); (2) hydrogenation of cinnamyl alcohol, where Qui *et al*.^11^ demonstrated higher conversion and selectivity using carbon-nanotube-supported Pd/Ru catalyst, as compared to that of either metal alone; (3) catalytic hydrogenation of levulinic acid; Luo *et al*.^12^ used a Pd/Ru bimetallic alloy where active NPs were a random dispersion with enhancement attributed to dilution and isolation of Ru by Pd, while Sykes & Stephanopoulos^13^ attributed selective hydrogenations to isolated Pd atoms; (4) methanol electro-oxidation; Pd-Ru bimetallic-NPs supported on carbon were reported by Monyoncho *et al*.^14^; (5) Hydrogen oxidation, where a Pd-overlayer enhanced the activity of Ru-nanotubes^15^. A ‘green’ application of these NPs is the conversion of 5-hydroxymethyl furfural (5-HMF) (a derivative of glucose or fructose)^16^ to 2,5-dimethyl furan (DMF)^17–19^, a ‘platform’ precursor of ‘drop in’ fuels and plastics. Ru catalyst converts 5-HMF to DMF but attempts to achieve higher yield and selectivity have focused on ‘classical’ mono/bimetallic catalysts^20–23^.

Here, we report the synthesis of novel bio-Ru and bio-Pd@Ru NPs assisted and supported/templated by cells of *B*. *benzeovorans*. We also illustrate, via a preliminary investigation, the potential of bio-Pd@Ru to convert 5-HMF to 2,5-DMF. These bio-derived catalyst systems facilitated a successful hydrogen transfer reaction enabling good yield under relatively low-pressure, low temperature (260 °C) and short reaction time (2 hours) conditions, which are comparable to currently reported processes using traditional mono/bimetallic catalysts^19,24^.

Furthermore, this work highlights the economic and biomass waste recycle potential associated with the synthesis and use of bacterial cells as a fabricator and support for Ru- and Pd/Ru NPs while cellulosic material, abundant in waste biomass, yields 5-HMF during thermochemical degradation^25^. Gram-negative *E*. *coli* cells can be sourced for economic NP-catalyst production as a waste by-product from another biotechnology process^26^, while Gram-positive *Bacillus* cells are generated on a large scale as waste during the commercial production of enzymes.

## Results and Discussion

### Deposition of Ru and Pd/Ru by cells of *Bacillus benzeovorans*

Previous work using Pd(II) showed its rapid, complete removal from solution by *B*. *benzeovorans* (to 5 or 20% of the cell dry weight), and conversion into Pd(0)-NPs at the cell surface and intracellularly within 30 min^7^ (Supplementary Information Fig. S2). In the current study Ru(III) was removed slowly (~96 h) and incompletely. Bimetallics were made by reducing Pd(II) on/in the cells as before (above) but using a lighter ‘seeding’ of Pd (Table 1) and then adding Ru(III) under H_2_. Either by providing Ru(III) alone or following seeding with Pd(0), ~40% of the Ru(III) remained in solution by assay suggesting an equilibrium position; the nominal and actual compositions of biomaterials used in this study are shown in Table 1.

**Table 1:** Materials examined in this study prepared on cells of *Bacillus benzeovorans*.

Sample Material		Nominal metal loading		Actual metal loading*		
		(wt%)		(wt%)		(Total wt%)
		Pd	Ru	Pd	Ru	
I	20 wt% bio-Ru	0	20%	0	14.2%	14.2%
II	20 wt% bio-Pd/Ru	10.0%	10.0%	9.7%	6.0%	15.7%
III	5 wt% bio-Pd/Ru	2.5%	2.5%	2.4%	2.0%	4.4%
IV	5% bio-Ru	0	5%	0	ND	ND
V	5% bio-Pd	5%	0	5%	0	5%

*The actual metal loading was determined by difference from the Ru(III) provided and that found in the spent solution by assay. Residual Ru(III) was determined in the spent solution by assay using stannous chloride. Ru(III) sample (0.2 ml, aq.) was added to 0.8 ml of stannous chloride (29.9 g SnCl_2_ in 500 ml conc. HCl) and incubated at 30 °C (30 min). Ru(III) was estimated at A_400_ with reference to a Ru(III)-calibration similarly determined and was linear in the region of interest. More than 95% of the Pd was removed in the pre-loading step (negligible residual Pd (II) was found by assay). Materials are referred to by their nominal metal loadings (5 wt % or 20 wt%) in this work. ND: Not determined.

**Figure 1:**
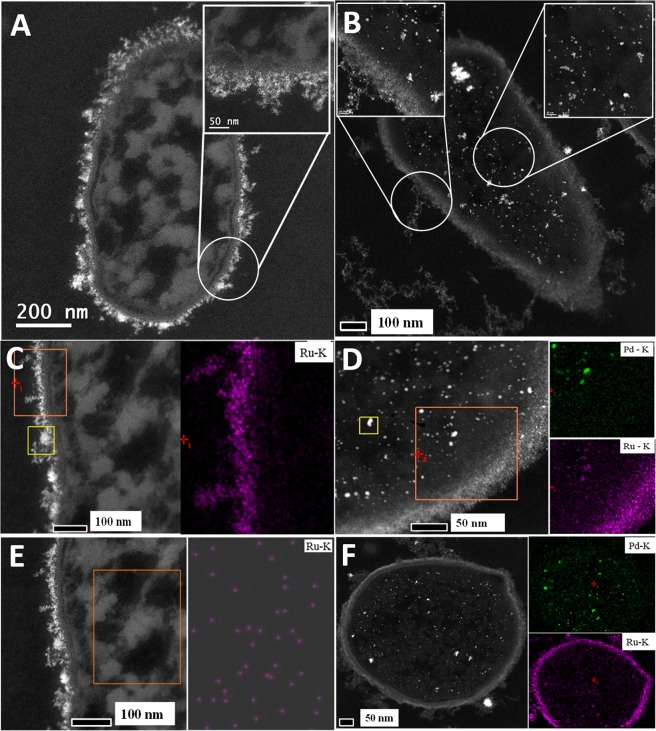
STEM images of sections of *B*. *benzeovorans* cells loaded to (nominally: see Table 1) 20 wt% bio-Ru (**A**,**C**,**E**) and 20 wt% bio-Pd/Ru (**B**,**D**,**F**). Left panels: backscattered electron images. Right panels: elemental maps for Pd and Ru as shown for cell surface and intracellular areas. Magnifications are as shown.

Cells without added metal showed no electron opaque deposits (Supplementary Information Fig. S3) while those challenged with Ru(III) alone showed cell surface-localized electron opaque material. Where Pd(0) had been pre-deposited, additional intracellular metallic deposits were visible (Supplementary Information Fig. S3). Elemental analysis of cell sections confirmed Ru-NPs, restricted to the cell surface of the former (Fig. 1A,C,E), while the bimetallic preparation showed intracellular NPs with co-localized Pd and Ru (Fig. 1B,D,E; Supplementary Information Fig. S4).

Like Pd, Ru has no known biological function. There is little information on the interactions of Ru^3+^ with living cells but Ru-complexes are very common, e.g. ruthenium-amine complexes are reported to have antitumor activity^27^ while a recent report^28^ describes the formation of Ru(III) complexes with collagen. In bacteria it seems likely that incoming Ru(III) is intercepted by amine groups of the n-acetyl glucosamine component of the thick peptidoglycan layer that characterizes the Gram-positive bacterial cell wall^29^. Extensive deposition of intracellular Pd ‘seeds’ was confirmed, with some nanostructures visible; relatively little Pd was apparent in the cell wall (Supplementary Information Fig. S2) although previous work using XPS confirmed the presence of Pd in the wall layers with coordination to nitrogen groups^30^. Hence, potential ligand sites for coordination of incoming Ru(III) may have been already occupied by Pd in the bimetallic, allowing more incoming Ru(III) to enter the deeper layers of the cell without interception. On the other hand, the relative paucity of wall-localized Pd following Ru(III) addition (Supplementary Information Fig. S4) may suggest that incoming Ru(III) may be able to displace Pd(II) to promote internalization of the latter.

### Examination of bio-Pd/Ru material by HRTEM, HAADF and X-ray powder diffraction

**Figure 2:**
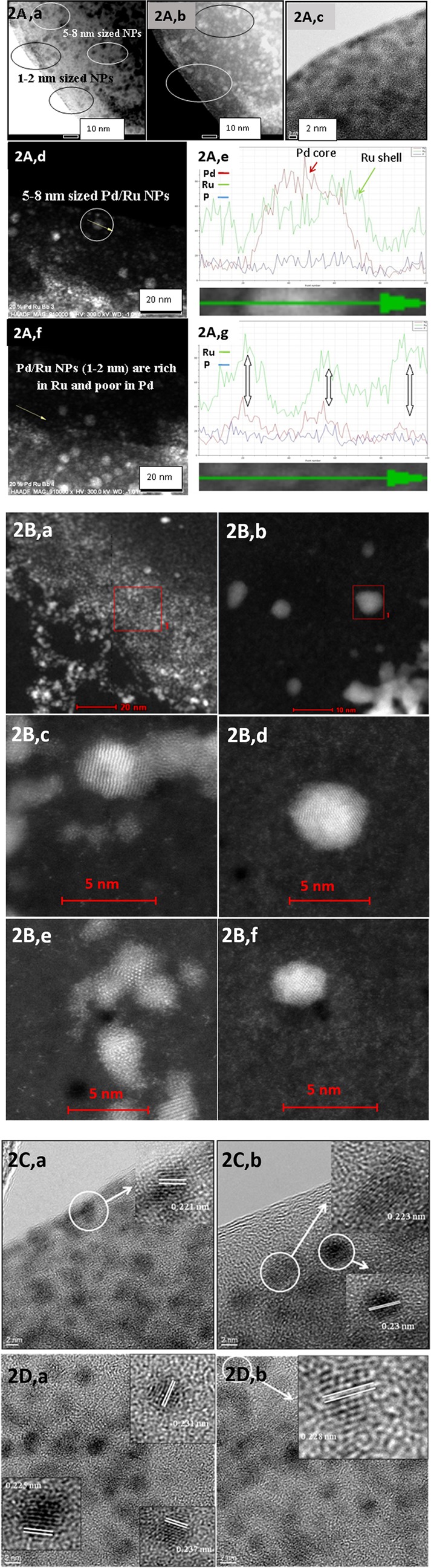
(**A**) Examination of bio-derived Pd/Ru (nominally 20 wt%) using STEM-HAADF (a,c) and via use of backscattered electrons (b). (d) Large nanoparticle with transect shown in e. Note core-shell structure. (f): Small nanoparticles with transect shown in (g). Note paucity of Pd. (**B**) HAADF-HR-STEM micrographs of cell surface Pd/Ru nanoparticles (a,c,e) and intracellular Pd/Ru nanoparticles (b,d,f) revealing lattice spacing in crystals and also the Pd and Ru atoms (e & f). (**C**) High resolution TEM micrographs of surface-localized Pd/Ru nanoparticles revealing lattice spacing in crystals. Bars are 2 nm. (**D**) High resolution TEM micrographs of intracellular Pd/Ru nanoparticles revealing lattice spacing in crystals. Bars are 2 nm.

Examination of a portion of a cell by energy dispersive X-ray analysis (EDX) confirmed the co-localization of Pd and Ru in selected areas but the NPs were not well-defined (Supplementary Information Fig. S5). Examination by high resolution TEM (Fig. 2A; a–c) using electron backscattering to facilitate metal visualization (Fig. 2A; d,f) shows nanostructures comprising very small NPs (1–2 nm) and other relatively larger NPs of 4–8 nm. A size distribution analysis was not done due to the difficulty of setting the NP boundaries and of visualizing the smallest NPs. Tyo & Vajda^31^ noted that small clusters can have unique and often unexpected properties; they have a high ratio of surface to bulk atoms, can share electrons differently from bulk materials and, being located at the cluster/support interface, the support can stabilize clusters in ‘wells’ and also provide steric hindrance. The proportion of Pd and Ru in the small bio-NPs was not measured but they contained both metals, as shown in a transect via EDX (Fig. 2A; f,g). However the small NPs were Pd-deficient with respect to Ru with a low background of phosphorus attributed to the supporting cell wall materials (Fig. 2A, f,g; Supplementary Information Fig. S2). Kyriakou *et al*.^32^ and Lucci *et al*.^33^ showed that single isolated Pd atoms are required for hydrogen activation in catalysis, via lowering the energy barrier to promote hydrogen dissociation and its spill over onto the surrounding metal terrace, which is key to hydrogenation selectivity^33^. An example bimetallic, Pd6Ru6 encapsulated in mesoporous silica, was tenfold more active as a selective hydrogenation catalyst than corresponding monometallics^10^.

A transect of a larger NP shows a core-shell structure comprising a Pd-enriched core and Ru-shell (Fig. 2A; d,e). This contrasts with bio-Au@Pd core-shells (Au core/Pd shell^4^) formed galvanically at the expense of Pd(0) ‘seeds’. Although Ru was dispersed throughout the NP, as well as at the core, (Fig. 2A; e) the area surrounding the NP was Ru-deficient, suggesting ‘recruitment’ of Ru atoms from the matrix into a Ru-shell. It is possible that the initial Pd-‘seeds’ must attain a critical size (or be held within a particular biochemical ‘well’) to exert an effect on the nearby Ru and promote core-shell formation. Cells loaded to 20 wt% with Pd alone also show larger NPs as well as very small ones (Supplementary Information Fig. S2) which could argue for ‘productive’ and ‘non productive’ cellular sites with respect to additional metal deposition. This heterogeneity was apparent also in the deposition of Au@Pd-NPs (Supplementary Information Fig. S1).

HAADF-HRSTEM revealed details of the surface-localized and cytoplasmic NPs (Fig. 2B). Z-imaging, where the image intensity reflects the Z dependence on atomic number, can be used to localize atoms in NPs where elements of higher atomic number appear brighter, e.g in bio-Au@Pd the Au-core was evident^6^ (Z Pd = 46; Z Au = 79) and similarly the Pt in Pd/Pt alloy (Z Pt = 78)^34^. However since Z Ru = 44 (i.e. close to Pd) the difference in contrast between the metals would be small and, accordingly, the core-shell structure was not well-defined (Fig. 2B; d). The contrast is also a function of the sample thickness (i.e. thicker at the NP centre) as well as other factors^35^. Examination of both types of NP (cell surface-localized and intracellular) by HRTEM imaging (Fig. 2C,D) gave a lattice spacing of ~0.231 nm, which corresponds to the (110) plane of RuO_2_^36^ whereas the Pd(111) facet in bio-Pd of *B*. *benzeovorans* was 0.250 nm^7^. From this, and Fig. 2A, e we suggest that the larger NPs in Sample II comprise a Pd-core surrounded by a Ru-layer which contains crystalline RuO_2_, i.e. an oxidized form of Ru as compared to the supplied Ru(III). No precaution was taken to exclude air (following H_2_-sparging), hence it would seem that Ru(III) becomes oxidized to Ru(IV) and forms RuO_2_ in the NPs. Examination of the bulk material by X-ray powder diffraction (Supplementary Information Fig. S6) shows no detectable crystalline component of cells with Ru-alone (the Ru appears as an ill-defined darkening of the cell surface: Supplementary Information Fig. S3), while the powder pattern of Pd(0) is identical with and without added Ru (nominally 5% Pd, 20% Ru: Supplementary Information Fig. S6) with no shift in peak positions. In contrast to the sequential metal additions reported here a Pd-Ru bimetallic was made via simultaneous reduction on a carbon support, showing a small shift of the Pd(220) peak, which suggested lattice contraction of Pd fcc by Ru and hence some alloying but the previous work had suggested alloying to be low^37^.

### Examination of cell surface bio-Ru and bio-Pd/Ru by X ray photoelectron spectroscopy

**Figure 3:**
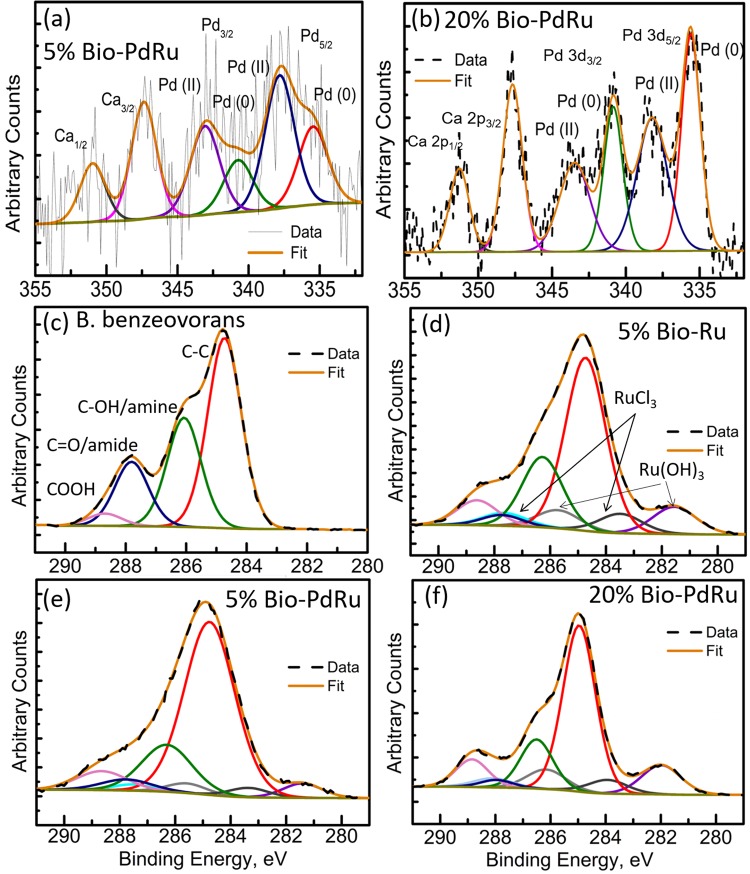
XPS analysis showing high resolution spectra for: (**a**) Pd3d for 5 wt% bio-derived Pd/Ru with fitted components; (**b**) Pd3d for 20 wt% bio-derived Pd/Ru with fitted components; (**c**) C 1s for *B*. *benzeovorans* with resolved components (**d**) C 1s for 5 wt% bio-derived Ru with resolved components; (**e**) C 1s for 5 wt% bio-derived Pd/Ru with resolved components and; (**f**) C 1s for 20 wt% bio-derived Ru with resolved components.

Next, the surface-bound NPs of whole cells (the outermost ~10 nm of the cell wall) were examined by XPS, where the reduction of Pd(II) to Pd(0) in the ‘seeding’ step was confirmed previously^30^. The wide energy spectrum for all samples is shown in Supplementary Information Fig. S7 compared with *B*. *benzeovorans* as well as Ru deposited on C. All samples clearly evidenced the presence of the C 1s and C 1s + Ru 3d peak along with the oxygen O 1s signal centred at ~285 and ~530 eV, respectively. Apart from these, the N 1s, nitrogen signal was evident in the *B*. *benzeovorans* samples. A lower intensity signal for the same was also seen in the bio-Pd/Ru and bio-Ru samples suggesting a possible decrease in the nitrogen content of the surface layer of the bacterial cells. A possible shedding of S-layer proteins from the cell wall as a response to metal-stress was not examined but the typical response of *Bacillus* spp. is to sorb platinum group metals onto the S-layer protein^38^. The presence of Pd was identified in high-resolution spectra (Fig. 3). The spectrum for commercial RuCl_3_ salt (the starting material) is shown in Supplementary Information Fig. S8, confirming Ru(III) as RuCl_3_ and Ru(OH)_3_ species.

High-resolution Pd 3d spectra for 5% bio-Pd/Ru and 20% bio-Pd/Ru samples are shown in Fig. 3a,b, respectively. The spectra were fitted using Voigt (mixed Gaussian-Lorentzian) peaks to identify the oxidation states of Pd. In both samples, Pd was found in its native Pd(0) and oxidized Pd(II) state (Table 2)^39,40^. However, as seen from the higher intensity Pd(0) peaks in the Pd 3d doublet in Fig. 3b as opposed to Fig. 3a the Pd(0): Pd(II) ratio was higher in the 20% bio-Pd/Ru than in the Pd 3d doublet in the 5% bio-Pd/Ru sample (also see Table 2). This suggests that the presence of a higher amount of Ru ‘safeguards’ Pd from being oxidized during the synthesis process despite a high amount of oxygen presence in the biomass reaction process. The presence of calcium, which is ubiquitous in biomass, was also confirmed here, the binding energies of Pd 3d and Ca 2p being close to each other.

**Table 2:** Speciation of Ru and Pd oxidation states in cell surface layers and elemental compositions, as determined by XPS.

Ruthenium	Ratio of Ru (III) to Ru (VI)			
	From 3d_5/2_			From 3p_3/2_
RuCl_3_ standard	—			8.29
5% Ru on C	1.76			1.76
5% bio-Ru	1.40			1.40
5% bio-Pd/Ru	1.55			1.55
20% bio-Pd/Ru	2.15			2.15
**Palladium**	**Ratio of Pd(0) to Pd(II)**			
	**From 3d**_**3/2**_			**From 3d**_**5/2**_
5% bio-Pd/Ru	0.59			0.59
20% bio-Pd/Ru	1.01			1.01
**Sample**	**% of total composition**			
	**O**	**C**	**Ru**	**Pd**
5% Ru on C^a^	11.9	79.4	5.4	—
5% bio-Ru	23.9	67.4	3.7	—
5% bio-Pd/Ru^b^	23.0	69.6	1.6	0.1
20% bio-Pd/Ru^c^	24.1	68.4	4.3	0.2

^a^Commercial catalyst; used ‘as provided’. ^b^Ruthenium loading was calculated as 2% by chemical assay (Table 1). ^c^Ruthenium loading was calculated as 6% by chemical assay (Table 1). Note that XPS analyses only the outermost ~10 nm of the sample, i.e. the bacterial cell wall layers. Percentage ‘underestimation’ was 20% and 28% respectively, denoting Ru located below the depth of analysis.

Figure 3c–f show the C 1s + Ru 3d high-resolution spectra with their components for *B*. *benzeovorans* and the three metal NP-loaded samples. The high-resolution C 1s spectra for *B*. *benzeovorans* (Fig. 3c) were resolved into four components identified as C-C (284.5 eV), C-OH/amine (286 eV), C=O/amide (288 eV) and COOH (290 eV), respectively. With the introduction and growth of metal NPs in the bacterial biomass (Fig. 3d–f) a significant change is evident in the spectra, confirming the loss of C=O/amide and C-OH/amine groups and the introduction of Ru components. This behaviour is very similar to the reduction of graphene oxide sheets upon nucleation of metal nanoparticles on them, suggesting oxygen functionalities present in the biomass being replaced by NPs during the nucleation process. This evidences the change in the state of carbon present in the biomass similar to a carbon support used in many electrocatalytic systems. A similar behaviour resulting in graphitization of biomass was reported by Priestley *et al*.^40^. Ru doublet components^41^ were found in the form of RuCl_3_ and Ru(OH)_3_ in all three metal NPs -bacterial biomass samples (Fig. 3d–f). The amount of Ru component appears to be higher in the 5% bio-Ru sample (Fig. 3d) than in the 5% bio-Pd/Ru sample which is attributed to the nominally 5 wt% of bio-Pd/Ru sample (Fig. 3e) comprising ~50% each of Pd and Ru (Table 1). The high-resolution O 1s spectra for all samples and the C 1s + Ru 3d spectrum for the RuCl_3_ reference sample are shown in Fig. S9. Specifically, the O 1s spectrum for 5 wt% bio-Pd/Ru shows a very small component peak at ~529.5 eV, suggesting a small amount of metal oxide being present. No such distinct component was resolved in the high-resolution O 1s spectra for 20 wt% bio-Pd/Ru. This is in good agreement with the observations from Fig. 3(a,b) confirming more metallic content in 20 wt% bio-Pd/Ru.

None of the spectra provide evidence for the occurrence of Ru(0) as no peaks are visible at 280 eV (the energy for Ru(0) in the Ru 3d_5/2_ orbital), or 461.5 eV (the energy for Ru(0) in the Ru 3p_3/2_ orbital) (Fig. 3d–f and Supplementary Information Figs S7 and S8). Previous reports suggest that in Pt-Ru catalysts the Ru is present as RuO_2_ and RuO_2_.xH_2_O (i.e. Ru(IV))^42,43^. However the NIST XPS database would suggest that in the present study the species at the cell surface are Ru(III) and Ru(VI). No precautions were taken to exclude air from the samples before analysis. The RuCl_3_ reference material showed ~90% of Ru(III) (Supplementary Information Fig. S8). The 5 wt% metal samples appeared to be enriched with respect to Ru(III) as compared to Ru(VI) with a possible enhancement in the proportion of Ru(III) (~40%) in the more heavily loaded bio-Pd/Ru sample (which has twice the Pd loading in the cell surface layers) (Fig. 3) suggesting a slight overall reduction via Pd oxidation in accordance with the increased proportion of Pd(II) (Table 2). However, the caveat of this conclusion is that, as shown in Supplementary Information Fig. S2, a significant amount of Pd was internalized during the pre-loading step (depleting the surface pool), which accounts for the low amounts of Pd visualized by XPS (Table 2). Since XPS is a surface technique (5–10 nm depth) accurate mass balances are not possible via this method.

### Analysis of bulk material using EXAFS: XANES Analysis

XANES provides information on the average oxidation state of metals such as Pd and Ru. Small shifts (a few eV) in XANES absorption edge energies can occur when a metal changes its average oxidation state.

The Supplementary Information (Fig. S10) shows the XANES regions of the EXAFS spectra obtained with palladium foil (metallic Pd), PdO (Pd(II)), and bio-Pd/Ru NPs (5 wt% and 20 wt% Pd-Ru). Comparison of the experimental spectra to the reference spectra clearly shows that Pd is present as a mixture of Pd(0) and Pd(II) in the two Pd/Ru samples. To determine the relative amounts of Pd(0) and Pd(II) present in the biologically-derived samples, we applied linear combination fitting mode of ATHENA code. The calculation revealed a mixture of 45% metallic palladium and 55% Pd(II) for both bulk bio-derived Pd/Ru NPs samples. This is in agreement with the XPS surface analysis of the 5% Pd/Ru sample but disagrees with the ~two-fold greater amount of reduced Pd(0) found at the surface in the 20% Pd/Ru sample (Table 2), suggesting that more of the Ru(III) oxidation reaction occurs in the surface NPs than in their intracellular bulk counterparts, i.e. in this case possible galvanic reduction of Pd(II) by Ru (III) in contrast to the earlier case with Pd/Au^4^ where Pd(0) was the reductant for Au(III). In each case the metal that was oxidized goes on to form the shell (c.f. Fig. 2A; e).

In the case of the Ru edge (Supplementary Information Fig. S11), the XANES spectra of both bio-NPs samples are different from that of Ru foil indicating that these bulk samples, like the surface NPs (above) do not contain metallic Ru. In this bulk sample, Ru is present as a mixture of Ru(III) and Ru(IV), whereas XPS (above) indicates Ru(VI), i.e. a more predominantly oxidized form of Ru in the surface in contact with air. The surface layer volume accessed by XPS (outermost 5–10 nm) is a small fraction of the total cell volume (see Fig. 1); hence Ru(VI) from the former would be below the limit of detection in the bulk sample in XANES. A mixed Ru(IV) and Ru(VI) was reported in nanocrystalline ruthenium oxide made on graphene flakes via microwave plasma chemical vapour deposition^36^.

### EXAFS: Pd K-edge

**Figure 4:**
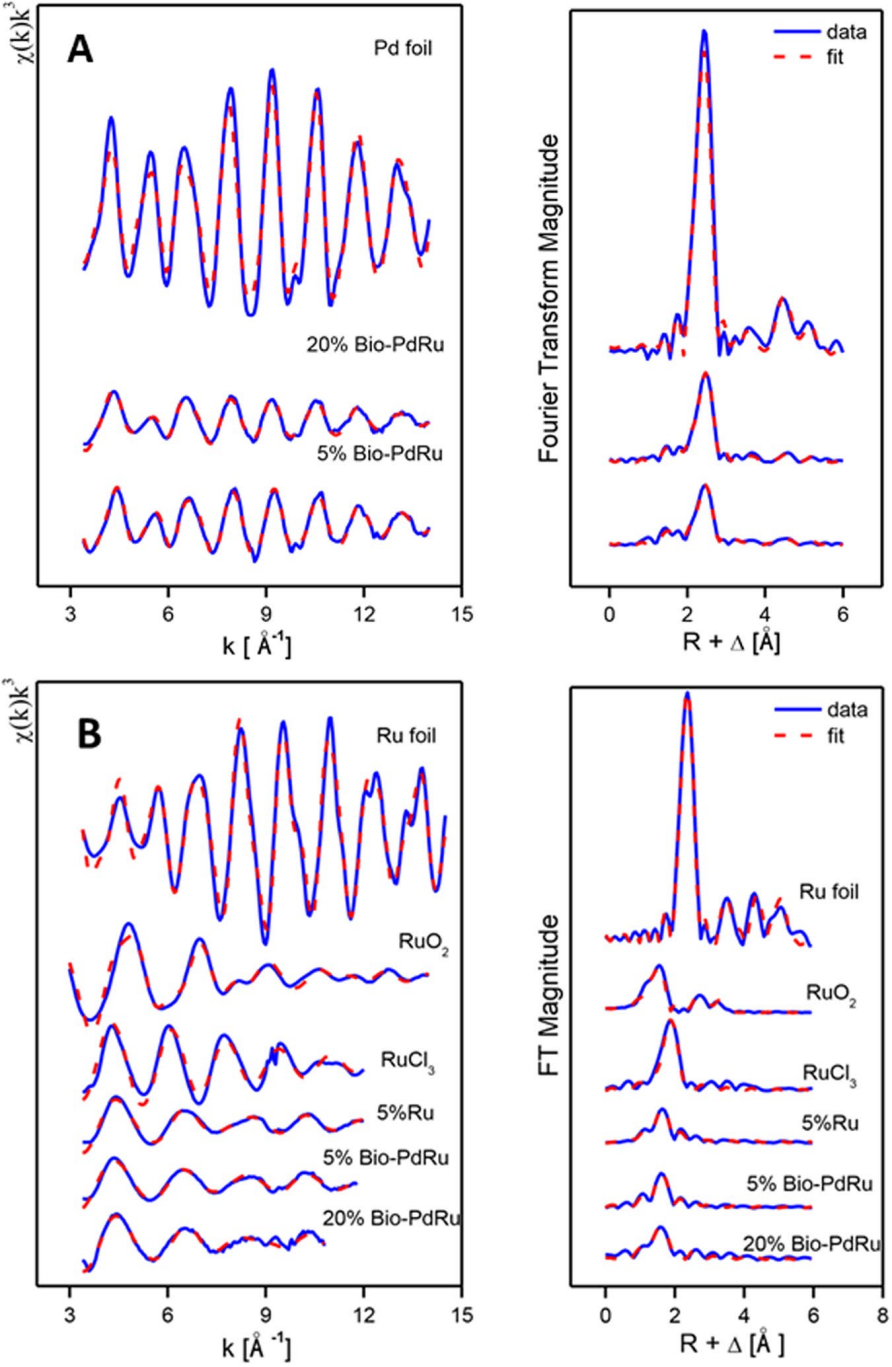
(**A**) *K*^3^-weighted EXAFS spectra of bio-derived Pd-Ru NPs and reference samples and their corresponding Fourier Transforms at Pd edge. (**B**) *K*^*3*^-weighted EXAFS spectra of bio-derived Pd-Ru NPs and reference samples and their corresponding Fourier Transforms at Ru edge.

The Pd K-edge EXAFS spectra of a palladium foil, and of 5 wt% and 20 wt% bio-Pd-Ru samples along with their corresponding Fourier transforms (FT) are shown in Fig. 4A. The fit parameters of the calculated spectra are given in Table 3. It is well established that the FT of χ(*k*) over a finite *k* range is a radial structure function exhibiting a series of peaks whose positions and magnitudes are related to the interatomic distances and the number of atoms in the different coordination shells, respectively. FT peak distances are reported in units of Å and are uncorrected for scattering phase shift, i.e., R + ΔR.

**Table 3:** EXAFS structural parameters of palladium foil and biogenic Pd-Ru NP samples and ruthenium foil, RuO_2_, RuCl_3_ and biogenic Ru and Pd-Ru NPs samples.

Sample	Shell	N^a^	R[Å]^b^	σ^2^ [Å^2^]^c^	ΔΕ[eV]
Pd foil	Pd-Pd_1_	12^d^	2.74	0.0047	−0.66
	Pd-Pd_2_	6^e^	3.86	0.0086	
	Pd-Pd_3_	24^e^	4.78	0.0083	
	Pd-Pd_4_	12^e^	5.4	0.0055	
5% Bio-PdRu	Pd-O	1.3 ± 0.3	2.05	0.0078	15.7
	Pd-Pd_1_	4.0 ± 0.4	2.74	0.0074	−4.5
	Pd-Pd_2_	2^e^	3.83	0.012	
	Pd-Pd_3_	8^e^	4.8	0.015	
	Pd-Pd_4_	4^e^	5.38	0.012	
20% Bio-PdRu	Pd-O	0.9 ± 0.2	2	0.0074	8.85
	Pd-Pd_1_	5.5 ± 0.4	2.74	0.007	−0.21
	Pd-Pd_2_	2.75^e^	3.84	0.011	
	Pd-Pd_3_	11.0^e^	4.79	0.015	
	Pd-Pd_4_	5.5^e^	5.4	0.012	
Ru foil	Ru-Ru_1_	12^d^	2.67	0.004	−1.81
	Ru-Ru_2_	6^d^	3.78	0.0028	
	Ru-Ru_3_	24^d^	4.68	0.0084	
	Ru-Ru_4_	12^d^	5.35	0.0031	
RuCl_3_	Ru-Cl	5.3 ± 0.3	2.35	0.0059	−2.7
RuO_2_	Ru-O_1_	2^d^	1.87	0.002	1.80
	Ru-O_2_	4^d^	1.99	0.002	
	Ru-Ru_1_	2^d^	3.09	0.0068	
	Ru-Ru_2_	8^d^	3.56	0.016	
5%Ru	Ru-O_1_	0.8 ± 0.1	1.87	0.0021	−1.61
	Ru-O_2_	3.3 ± 0.2	2.05	0.0021	
	Ru-Ru	0.5 ± 0.1	2.85	0.005^d^	
5% bio-PdRu	Ru-O_1_	0.8 ± 0.1	1.87	0.0021	−1.61
	Ru-O_2_	3.3 ± 0.2	2.05	0.0021	
	Ru-Ru	0.5 ± 0.1	2.85	0.005^d^	
20% bio-PdRu	Ru-O_1_	1.0 ± 0.1	1.89	0.002	−2.08
	Ru-O_2_	3.1 ± 0.1	2.06	0.002	
	Ru-Ru	0.6 ± 0.1	2.79	0.005^d^	

^a^Errors in coordination numbers are ±25% and standard deviations as estimated by EXAFSPAK; ^b^Errors in distance are ±0.02 Å; ^c^Debye-Waller factor; ^d^Fixed for calculation, ^e^Coordination number (N) linked to the N of Pd-Pd_1_ path.

In the case of the Pd foil, the FT peaks of metallic Pd were attributed to four Pd-Pd shells with distances of 2.74, 3.86, 4.78, 5.40 Å. The major peak corresponds to about twelve Pd atoms at a Pd-Pd interatomic distance of 2.74 ± 0.02 Å as reported by Polizzi *et al*.^44^.

In the 5 wt% and 20 wt% bio-Pd/Ru samples, the Pd is present in two different phases, one metallic (Pd-Pd) and one complexed (Pd-O) via oxygen atoms to the template cell matrix. For the Pd-O phase, the distances found are comparable to the ones of palladium oxide having a simple tetragonal structure^45^ with Pd-O contributions at 2.0 ± 0.02 Å and 2.05 ± 0.02 for the 20 wt% and 5 wt% Pd-Ru samples, respectively. The distance was identified using the Pd–O backscattering phase and amplitude functions obtained from atomic coordinates of PdO using the FEFF 8 program. The oxygen atoms could be arising from the carboxyl groups of the aspartic and glutamic acids of the cells^38^. For the metallic phase, the interatomic distances found are very close to the ones of the metallic foil. For the 20%Pd-Ru NPs, the intensity of the first shell Pd–Pd scattering is lower than that of Pd foil, and higher than of 5%Pd-Ru. These results indicate that the bimetallic Pd/Ru NPs are smaller in the 5%Pd-Ru that those in the 20%Pd-Ru sample and that the size of the NPs in both bio-derived samples is smaller than that of bulk Pd foil.

### EXAFS: Ru K-edge

The Ru K-edge EXAFS spectra of a ruthenium foil, RuO_2_, RuCl_3_, 5% Ru and of 5 wt% and 20 wt% bio-Pd-Ru samples along with their corresponding Fourier transforms (FT) are shown in Fig. 4B. The structural parameters of the calculated spectra are summarized in Table 3.

The FT of the three experimental samples was well fitted by the use of two Ru-O bonds with interatomic distances of 1.87–1.90 and 2.05 ± 0.02 Å, and a single Ru-Ru shell with an interatomic distance of 2.80–2.85 ± 0.02 Å (Table 3).

The distance of the shortest Ru-O bond (1.87–1.89 ± 0.02 Å) can be assigned to Ru = O of the RuO_2_^46^ while the peak at bond distance of 2.05 Å is assigned to Ru-OH species as observed in RuHAP-γ-Fe_2_O_3_ (1.95 Å)^47^. The absence of Ru–Ru or Ru–Pd peaks confirm that atomically dispersed Ru is on the surface of the NPs in oxidized form.

### Catalytic activity of the metallized cells in conversion of 5-HMF to 2,5-DMF

The catalytic transfer hydrogenation of 5-HMF to 2, 5-DMF using cell-supported Pd/Ru bimetallic nanocatalysts was briefly examined under H_2_. Tests using the 20 wt% bio-derived Ru (14 wt% Ru: sample I, Table 1) gave 95% conversion of 5-HMF with a yield of DMF of 47.9 ± 2.0% which was increased to 60.3 ± 3.0% (at the same conversion) by incorporation of Pd to make the bimetallic (with 4% less Ru: 9.7 wt% Pd/6 wt% Ru: Sample II, Table 1). To check the potential of the bio-derived catalysts at a more economical metal loading, the loading of Pd/Ru was reduced to nominally 2.5 wt% each (Pd 2.4 wt%; Ru 2.0 wt%; Sample III in Table 1) giving a conversion of 94.7% with a yield of 55.5 ± 2.0% which was not significantly less than the catalyst made at the higher metal loading.

## Conclusions and Outlook

We report the biomanufacture of a novel bio-supported Pd/Ru bimetallic catalysts using a facile and scalable synthesis method and show its potential for catalytic transfer hydrogenation to upgrade a waste from biomass processing (5-HMF) into a ‘drop in fuel’ precursor (DMF). Two types of bimetallic bio-derived nanoparticles were formed comprising a Pd/Ru mixture in small (1–2 nm) NPs and also core-shells (Pd@Ru) in larger (4–8 nm) NPs. Using complementary methods Pd(0) and Pd(II) were found and also Ru (III), (IV) and (VI) but no Ru(0). No lattice fringes were evident for Pd(0) (although the bulk XRD pattern evidenced metallic Pd) but lattice fringes attributable to RuO_2_ were apparent in both types of NP, i.e. ruthenium oxide at the NP surface. Attribution of the catalysis to one or other type of NP was not attempted, nor the potential catalytic diversity explored. However, in contrast to physico-chemical synthesis, the tools of synthetic biology (e.g. gene deletion/overexpression, physiological controls during ‘seeding’), capable to modulate isolated steps in an orthogonal synthesis, are particularly well suited to ‘engineer’ bio-derived NP synthesis, on a ‘canvas’ of bio-support matrix of particular cell types.

## Methods

### Bacterial preparations and fabrication of monometallic and bimetallic bionanoparticles

*Bacillus benzeovorans* NCIMB 12555 was grown aerobically (180 rpm, 30 °C) in medium comprising (g/L): beef extract 1.0 (Sigma-Aldrich), yeast extract 2.0 (Sigma-Aldrich), peptone 5.0 (Sigma-Aldrich) and NaCl 15.0 in distilled water (pH 7.3)^7^ and harvested (OD_600_ of 0.7–1.0) by centrifugation (9,000 × g, 15 min, 4 °C), washed three times in air (20 mM MOPS-NaOH buffer, pH 7.0) and stored as a concentrated suspension overnight (4 °C). Cell dry weight was estimated from a previously-determined OD dry weight conversion.

### Preparation of monometallic and bimetallic bio-derived nanoparticles (bio-NPs)

For monometallic (bio-Ru; nominal wt% as specified) cell suspension was diluted into 2 mM Ru (III) (RuCl_3_.2H_2_O solution, pH 2; 30 min; 30 °C) to the required biomass/metal ratio. H_2_ was bubbled (~1 h) left saturated (sealed bottle; 180 rpm agitation; 30 °C; 96 h). Synthesis of bimetallic Pd/Ru used, sequentially, a 2 mM Pd (II) and a 1 mM Ru (III) solution according to^4^ with modifications: 2 mM Pd (II) solution was reduced to Pd(0) on the cells under H_2_ (30 min; with about 95% removal (by assay) of residual soluble metal)^30^. The bio-Pd was washed twice (distilled water) and then water (2:1 vol/wt) was added to the pellet with respect to the initial volume of Pd solution that was used for reduction. The resulting mixture (reduced Pd in water) was subsequently used as a template to reduce 1 mM Ru (III) (added volume as required) to give a final loading of 2.5 wt% Pd/2.5 wt% Ru or 10 wt% Pd/10 wt% Ru (nominal metal loadings- see Table 1). The actual metal loadings (Table 1) were estimated by difference from assay of residual soluble Ru^30^. The mixture was saturated with H_2_ (above; 180 rpm, 30 °C; 96 h). The presumptive bimetallic bio-NPs were washed three times (distilled water) and once with acetone (9,000 × g, 15 min, 4 °C), air- dried and ground manually.

### High resolution scanning-transmission electron microscopy (STEM) with HAADF (high-angle annular dark field) detector, energy dispersive X-ray analysis (EDX) and determination of lattice spacing

Where cell sections were to be examined fresh preparations were fixed and processed (Fig. S2). Samples were fixed (2.5% (w/v) glutaraldehyde fixative in 0.1 M cacodylate buffer, pH 7.2; 2 h; 4 °C), washed three times with the same buffer and stained (1% aq. osmium tetraoxide). For TEM thin samples were prepared^48^. Alternatively, dried catalyst preparations (above) were suspended in ethanol, sonicated briefly and dropped onto carbon coated copper TEM grids. Electron opaque deposits were examined by EDX with peaks sought corresponding to X-ray emission energies of Ru and Pd. STEM and EDX were done using a FEI image Cs-corrector configuration Titan^TM^ G2 60–300 STEM microscope equipped with HAADF detector, accelerating voltage of 300 kV. Lattice spacings were determined using “ImageJ” through profiling of high resolution HAADF-STEM images.

### X-Ray photoelectron spectroscopy (XPS)

XPS used an Al Kα source. Surface chemical composition and oxidation state analyses was done as described^30^. Data were collected using a Sphera electron analyser (Omicron Nanotechnology), with the core levels recorded using a pass energy of 10 eV (resolution approx. 0.47 eV). Due to the insulating nature of the samples, a CN10 charge neutralizer (Omicron Nanotechnology) was used to prevent surface charging. A low energy (typically 1.5 eV) beam of electrons was directed on to the sample during XPS data acquisition. Measurements were made at room temperature and at a take-off angle of 90°, allowing a maximum probing depth of approx. 5–10 nm to evaluate bio-NPs bound to the outermost cell surfaces. Generated data were converted into VAMAS format and analysed using the CasaXPS package^49^ employing Shirley backgrounds, mixed Gaussian-Lorentzian (Voigt) lineshapes and asymmetry parameters where appropriate. All binding energies were calibrated to the C 1s peak originating from C-H or C-C groups at 284.6 eV. Selected graphs were re-plotted using origin software for achieving better fitting and component identification.

### Extended X-ray absorption fine structure (EXAFS) analysis

Pd and Ru K–edge X-ray absorption spectra were collected at the Dutch-Belgian Beamline (DUBBLE) at the European Synchrotron Radiation Facility (ESRF), Grenoble (France) using a Si(111) monochromator operating in fixed-exit mode. The data were collected in transmission mode using Ar/He filled ionization chambers. The energies were calibrated by measuring the Pd and Ru K-edge transmission spectra of Pd and Ru foils and were calibrated to 24350 and 22117 eV, respectively. The Ru and Pd/Ru-loaded samples were measured as dry samples (powder). Data were processed by using the ATHENA code^50^. Background removal was performed by means of a pre-edge linear function. Atomic absorption was simulated with a square-spline function. The amplitude reduction factor was held constant at 1.0 for the FEFF8 calculation and EXAFS fits. The shift in threshold energy, ΔE0, was varied as a global parameter in the fits. The theoretical scattering phase and amplitude functions used in data analysis were calculated using FEFF8^51^. For the Pd edge EXAFS spectra, data for phase-shifts and backscattering amplitudes were obtained from the PdO (Pd–O scattering) and Pd foil (Pd–Pd scatterings) reference compounds. For the Ru edge EXAFS spectra, data for phase-shifts and backscattering amplitudes were obtained from the RuO_2_ (Ru–O scattering), Ru foil (Ru–Ru scatterings) and RuCl_3_ (Ru-Cl scattering) reference compounds.

### Conversion of 5-hydroxymethyl furfural to 2,5-dimethyl furan catalysed by bio-Ru and bio Pd/Ru

5-HMF (≥99%) and 2,5-DMF (99%) were from Sigma Aldrich. Catalytic transfer hydrogenation reactions used a 100 mL stainless steel reactor (Parr; external temperature and stirring controllers). For a typical reaction the reactor was charged with 0.05 M 5-HMF in tetrahydrofuran (25 mL) and 0.05 g catalyst, sealed, purged with H_2_ (5 bar) and charged with 50 bar H_2_. The reaction (260 °C; 2 h; 700 rpm) was stopped on ice (time as determined by prior tests) and the mixture was filtered (Whatman; 8 µm). 5-HMF and 2,5-DMF were analysed by GC Shimadzu 2010; FID detector and ZB-SemiVolatile column: 30 m × 0.25 mm × 0.50 µm; Waters GCT Premier time-of-flight mass spectrometer (Micromass, Manchester, UK) with a ZB-SemiVolatile-MS column; injection volume 1 µl; injector temperature 300 °C; detector temperature; 300 °C, inlet pressure 100 KPa; split ratio of 100:1, with He carrier gas at a flow rate of 1 mL/min). An initial column temperature (50 °C) was held for 5 min, the temperature was ramped (16.6 °C/min) to 133.3 °C, held for 1.5 min, ramped to 300 °C and held for 3.5 min. For GC-MS analysis, Waters MassLynx software (version 4.1) was used to operate the GC-TOFMS system. The source temperature was 200 °C and electron energy was 70 eV. 5-HMF conversion and DMF yield were based on standards. Other products were not quantified.

## Supplementary information

Supplementary information

## References

[1] Zaleska-Medynska A, Marchelek M, Diak M, Grabowska E (2016). Noble metal-based bimetallic nanoparticles: the effects of the structure on the optical, catalytic and photocatalytic properties. Adv. Colloid. Interface Sci..

[2] Singh, O. V. (Ed.). *Bio-nanoparticles Biosynthesis and Sustainable Biotechnological Implications Wiley*, (2015).

[3] Basiuk, V. & Basiuk, E. V. (Eds). *Green Processes for Nanotechnology; from Inorganic to Bioinspired Nanonomaterials*. (Springer 2015).

[4] Deplanche K (2012). Microbial synthesis of core/shell gold/palladium nanoparticles for applications in green chemistry. J. Roy. Soc. Interface..

[5] Hosseinkhani B (2012). Microbially supported synthesis of catalytically active bimetallic Pd-Au nanoparticles. Biotechnol. Bioeng..

[6] Tran DT (2012). Configuration of microbially synthesized Pd-Au nanoparticles studied by STEM-based techniques. Nanotechnol..

[7] Omajali JB, Mikheenkho IP, Merroun ML, Wood J, Macaskie LE (2015). Characterization of intracellular palladium nanoparticles synthesized by *Desulfovibrio desulfuricans* and *Bacillus benzeovorans*. J. Nanopart. Res..

[8] Omajali JB, Mikheenko IP, Overton TW, Macaskie LE (2019). Probing the viability of palladium-challenged bacterial cells using flow cytometry. J. Chem. Technol. Biotechnol..

[9] Yao Y (2016). Modulating fcc and hcp ruthenium on the surface of palladium-copper alloy through tunable lattice mismatch. Ang. Chemie.

[10] Raja, R. *et al*. Preparation and characterisation of a highly active bimetallic (Pd-Ru) nanoparticle heterogeneous catalyst. *Chem*. *Commun*. 1571–1572 (1999).

[11] Qui J (2006). Selective hydrogenation of cinnamaldehyde over carbon nanotube supported Pd-Ru catalyst. React. Kinet. Catal. Letts..

[12] Luo W (2015). High performing and stable supported nano-alloys for the catalytic hydrogenation of levulinic acid to gamma- valerolactone. Nature Comm..

[13] Sykes, C. & Stephanopoulos, M. F. Isolated Pd atoms allow highly selective catalysis of hydrogenation reactions US Dept of Energy science. energy.gov/bes/highlights/2014/bes-2014-06-d/ (2014).

[14] Monyoncho EA, Ntais S, Soares F, Woo TK, Baranova EA (2015). Synergetic effect of palladium-ruthenium nanostructures for ethanol electrooxidation in alkaline media. J. Power Sources.

[15] St John S (2015). Platinum and palladium overlayers dramatically enhance the activity of ruthenium nanotubes for alkaline hydrogen oxidation. ACS Catal..

[16] Van Putten RJ (2013). Hydroxymethylfurfural, A versatile platform chemical made from renewable resources. Chem. Rev..

[17] Lei H (2014). Selective transformation of 5-hydroxymethylfurfural into the liquid fuel 2,5-dimethylfuran over carbon-supported ruthenium. End. Eng. Chem. Res..

[18] Nagpure AS (2015). Renewable fuels from biomass-derived compounds: Ru-containing hydrotalcites as catalysts for conversion of HMF to 2,5-dimethylfuran. Catal. Sci. Technol..

[19] Zhang F, Liu Y, Yuan F, Niu X, Zhu Y (2017). Efficient production of the liquid fuel 2,5-dimethylfuran from 5-hydroxymethylfurfural in the absence of acid additive over bimetallic PdAu supported on graphitized carbon. En. Fuels.

[20] Hansen TS, Barta K, Anastas PT (2012). One-pot reduction of 5-hydroxymethylfurfural via hydrogen transfer from supercritical methanol. Green Chem..

[21] Zu YH (2014). Efficient production of the liquid fuel 2,5-dimethylfuran from 5-hydroxymethylfurfural over Ru/Co_3_O_4_ catalyst. Appl. Catal. B.

[22] Nishimura S, Ikeda N, Ebitani K (2014). Selective hydrogenation of biomass-derived 5-hydroxymethylfurfural (HMF) to 2, 5-dimethylfuran (DMF) under atmospheric hydrogen pressure over carbon supported PdAu bimetallic catalyst. Catal. Today.

[23] Luo J, Arroyo-Ramirez L, Gorte RJ, Tzoulaki D, Vlachos DG (2015). Hydrodeoxygenation of HMF over Pt/C in a continuous flow reactor. AICHE Journal..

[24] Shi J, Wang Y, Yu X, Du W, Hou W (2016). Production of 2,5-dimethylfuran from 5-hydroxymethylfurfural over reduced graphene oxides supported Pt catalyst under mild conditions. Fuel.

[25] Román-Leshkov Y, Barrett CJ, Liu ZY, Dumesic JA (2007). Production of dimethylfuran for liquid fuels from biomass-derived carbohydrates. Nature.

[26] Zhu J, Wood J, Deplanche K, Mikheenko IP, Macaskie LE (2016). Selective hydrogenation using palladium bioinorganic catalyst. Appl. Catal. B Environ..

[27] Lima, A. P. *et al*. Cytoxicity and apoptotic mechanism of ruthenium(II) amino acid complexes in sarcoma-180 tumor cells. *PLoS ONE*, 10.1371/journal.pone.0105865 (2014). 10.1371/journal.pone.0105865 PMC4201456 25329644

[28] Luo D (2018). Biomimetic organization of a ruthenium-doped collagen-based carbon scaffold for hydrogen evolution. J. Mat. Chem. A..

[29] Potekhina NV (2011). Phosphate-containing cell wall polymers of bacilli. Biochem. (Mosc)..

[30] Omajali, J. B. *Novel bionanocatalysts for green chemistry applications*. (PhD Thesis University of Birmingham, UK, 2015).

[31] Tyo EC, Vajda S (2015). Catalysis by clusters with precise numbers of atoms. Nature Nanotechnol..

[32] Kyriakou G (2014). Significant quantum effects in hydrogen activation. ACS Nano..

[33] Lucci FR (2016). Controlling hydrogen activation, spillover, and desorption with Pd–Au single-atom alloys. J. Phys. Chem. Lett..

[34] Esparza, R. *et al*. Study of PtPd bimetallic nanoparticles for fuel cell applications. *Mat*. *Res*. **20**, 10.1590/1980-5373-mr-2016-0934 (2017)

[35] Nellist PD, Pennycook SJ (2000). The principles and interpretation of annular dark field Z-contrast imaging. Adv. Imag. Elect. Phys..

[36] Soin N, Roy SS, Mitra SK, Thubdat T, McLaughlin JA (2012). Nanocrystalline ruthenium oxide dispersed few layered graphene (FLG) nanoflakes as supercapacitor electrodes. J. Mat. Chem..

[37] Wei M, Hsu C, Liu F (2016). One-pot synthesis of mixed-phase Pd-Ru/C as efficient catalysts for electro-oxidation of formic acid. Int. J Electrochem. Sci..

[38] Fahmy K (2006). Secondary structure and Pd(II) coordination in S-layer proteins from *Bacillus sphaericus* studied by infrared and X-ray absorption spectroscopy. Biophys. J..

[39] Liu X, Yu H, Scott K (2015). Preparation and evaluation of a highly stable palladium yttrium platinum core–shell–shell structure catalyst for oxygen reduction reactions. Appl. Catal. B Environ..

[40] Priestley RL (2915). Pd nanoparticles supported on reduced graphene- *E*. *coli* hybrid with enhanced crystallinity in bacterial biomass. RSC Adv..

[41] Morgan DJ (2015). Resolving ruthenium: XPS studies of common ruthenium materials. Surf. Interf. Anal..

[42] Rolison DR, Hagans PL, Swider KE, Long JW (1999). Role of hydrous ruthenium oxide in Pt–Ru direct methanol fuel cell anode electrocatalysts:  the importance of mixed electron/proton conductivity. Langmuir.

[43] Wang H (2016). Role of Ru oxidation degree for catalytic activity in bimetallic Pt/Ru nanoparticles. J. Phys. Chem..

[44] Polizzi S, Riello P, Balerma A, Benedetti A (2001). Nanostructure of Pd/SiO_2_ supported catalysts. Phys. Chem. Chem. Phys..

[45] Borowski, M. Size determination of small Cu-clusters by EXAFS *J*. *Phys*. *IV Franc*e **7**, C2-259–C2-260 (1997).

[46] McKeown DA (1999). Structure of hydrous ruthenium oxides:  Implications for charge storage. J. Phys. Chem. B..

[47] Mori K (2007). Development of ruthenium–hydroxyapatite-encapsulated superparamagnetic γ-Fe2O3 nanocrystallites as an efficient oxidation catalyst by molecular oxygen. Chem. Mater..

[48] Merroun ML (2005). Complexation of uranium by cells and S-layer sheets of *Bacillus sphaericus* JG-A12. Appl. Environ. Microbiol..

[49] Fairley, N. CasaXPS. Casa Software Ltd, www.casaxps.com (2013.).

[50] Ravel B, Newville M (2005). ATHENA, ARTEMIS, HEPHAESTUS: data analysis for X-ray absorption spectroscopy using IFEFFIT. J. Synchrotron Rad..

[51] Ankudinov AL, Ravel B, Rehr JJ, Conradson SD (1998). Real-space multiple-scattering calculation and interpretation of X-ray absorption near-edge spectra. Phys. Rev. B..

