# Supplementary material for: Novel catalytically active Pd/Ru bimetallic nanoparticles synthesized by Bacillus benzeovorans
Source: Sci Rep. 2019 Mar 18;9:4715. doi: 10.1038/s41598-019-40312-3 (PMC6423089; doi:10.1038/s41598-019-40312-3)
Supplement: Supplementary file 1 — Supplementary information [file 41598_2019_40312_MOESM1_ESM.pdf]

Supplementary information

Novel catalytically active Pd/Ru bimetallic nanoparticles synthesized by *Bacillus benzeovorans*

Jacob B. Omajali, Jaime Gomez-Bolivar, Iryna P. Mikheenko, Surbhi Sharma, Bayonle Kayode, Bushra Al-Duri, Dipanjan Banerjee, Marc Walker, Mohamed L. Merroun, Lynne E. Macaskie

Figure S1

Intracellular core-shell Pd/Au nanoparticles in *Escherichia coli*: an example of biogenic core-shell material (I. Mikheenko, M.L. Merroun and L.E. Macaskie unpublished)

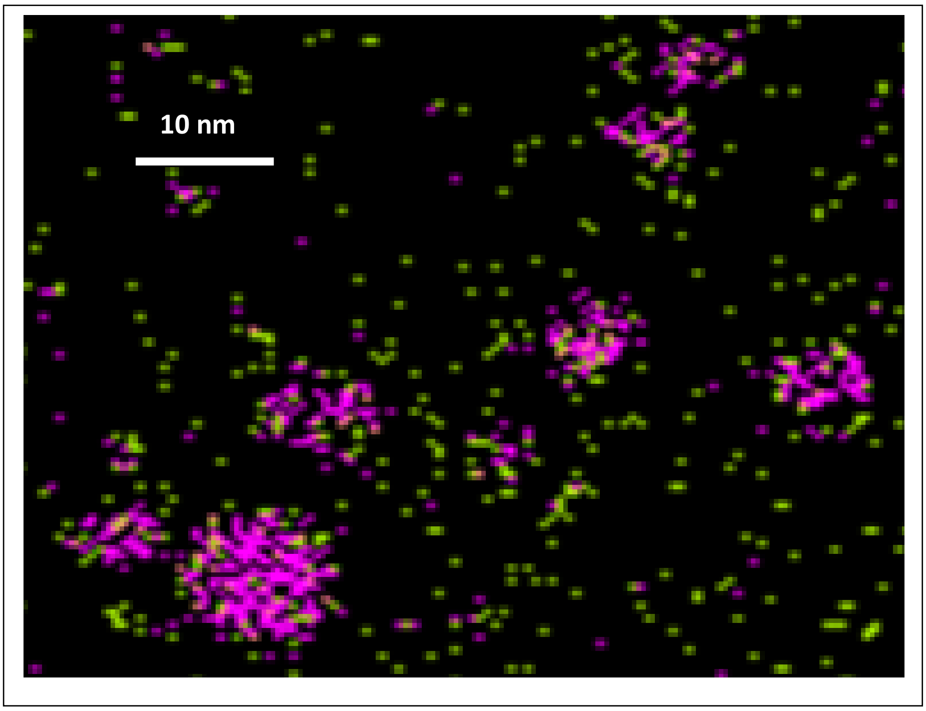

An area of the cytoplasm of *E. coli* is shown, loaded with 2.5wt% Pd initially and then 2.5 wt% Au by galvanic reduction of Au(III) catalysed by seeds of Pd(0)[4]. The image shows elemental mapping showing Au (magenta) and Pd (green). Metallised cells were viewed using high-resolution HAADF-STEM (High-Angle Annular Dark Field-Scanning Transmission Electron Microscopy) with EDX (Energy Dispersive X-ray Spectroscopy)[7], (see text). This is evidence that both Pd and Au are able to enter the bacterial cell and that Au localizes into discrete nanostructures where Pd was initially deposited. Since *Bacillus benzeovorans* also localises Pd(0)-NPs intracellularly (Fig. S2) this paradigm represents a possible intracellular arrangement of Pd/Ru, which was sought in the current work.

Figure S2. Intracellular Pd-nanoparticles in cells of *B. benzeovorans*

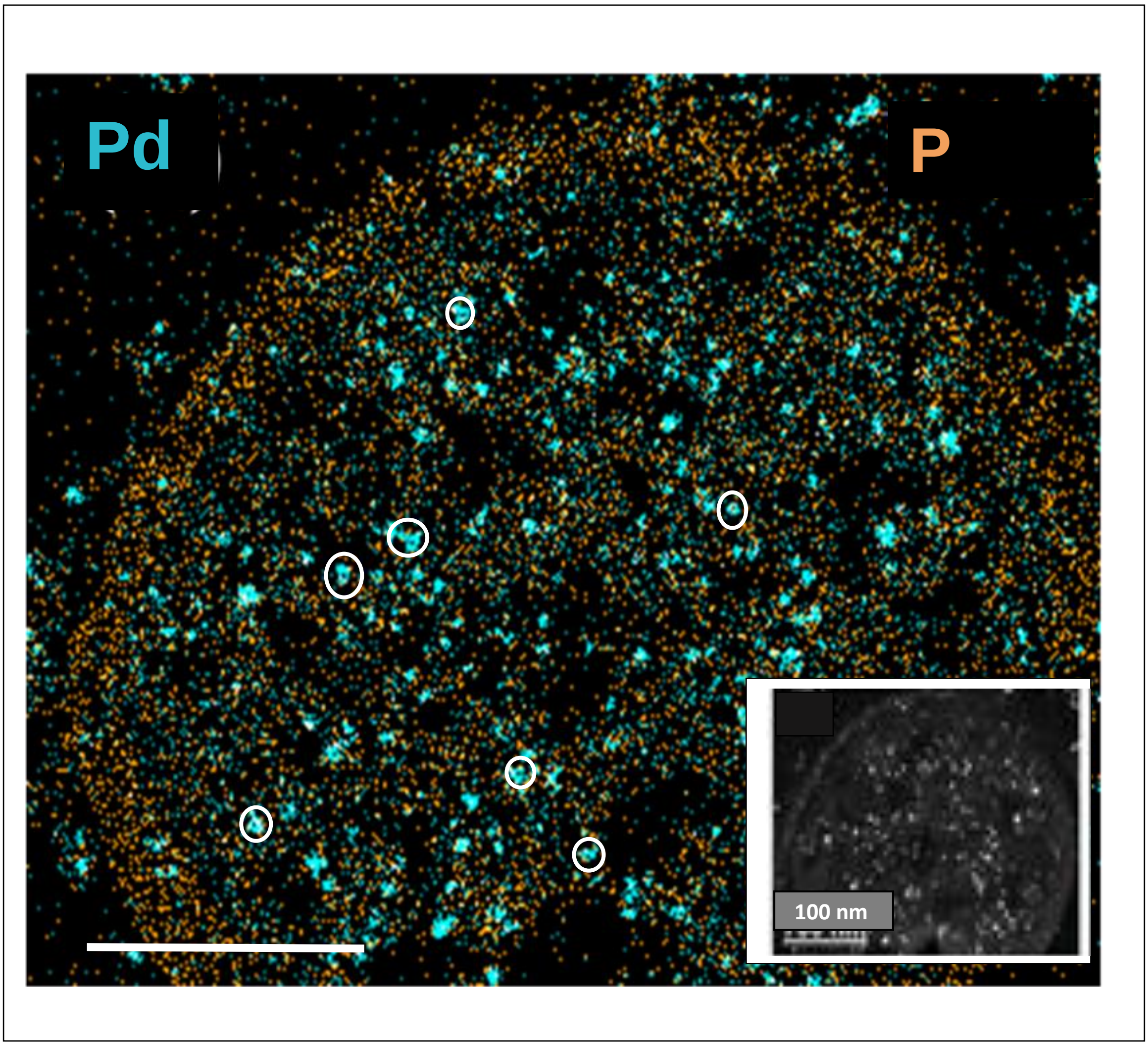

EDX mapping (from Image J) of *B. benzeovorans* (20 wt% Pd) shows co-localization of Pd (blue) with P (orange) within the cell cytoplasm. Note the relative lack of Pd at the cell surface; phosphate groups appear as orange and are attributed to the high content of teichoic acids typical of the Gram positive bacterial cell surface. Costes test for co-localization shows a correlation (R = 0.96) and Mander's overlap coefficient (R = 0.998), indicative of high co-localization between Pd and P[30]. Inset: TEM of cell with Pd-nanoparticles shown by electron backscattering As there is comparatively low Pd deposited in the cell wall it can be suggested that deposition of Pd is non-random (i.e. not simply by biosorption) and an uptake system for Pd is implied. Methodology: Bio-NPs on bacteria were washed (twice, distilled water), fixed (2.5% (w/v) glutaraldehyde in 0.1M cacodylate buffer, pH 7.0; at 4°C) and stained with 1% osmium tetraoxide. Preparations were dehydrated (ethanol series), embedded (epoxy resin) and sectioned (100-150 nm). At 5% metal loading metallic nanoparticles are indistinct. Hence, for electron microscopy higher metal loadings (20%) of the bio-NPs were examined. Note small donut-shaped structures (circled; diameter was ~ 2-3 nm). Note also that the cross-sectional diameter of the DNA helix is also 2 nm but no attempt was made to ascertain if the phosphate groups on DNA were Pd-binding sites. Bars are 100 nm.

Figure S3

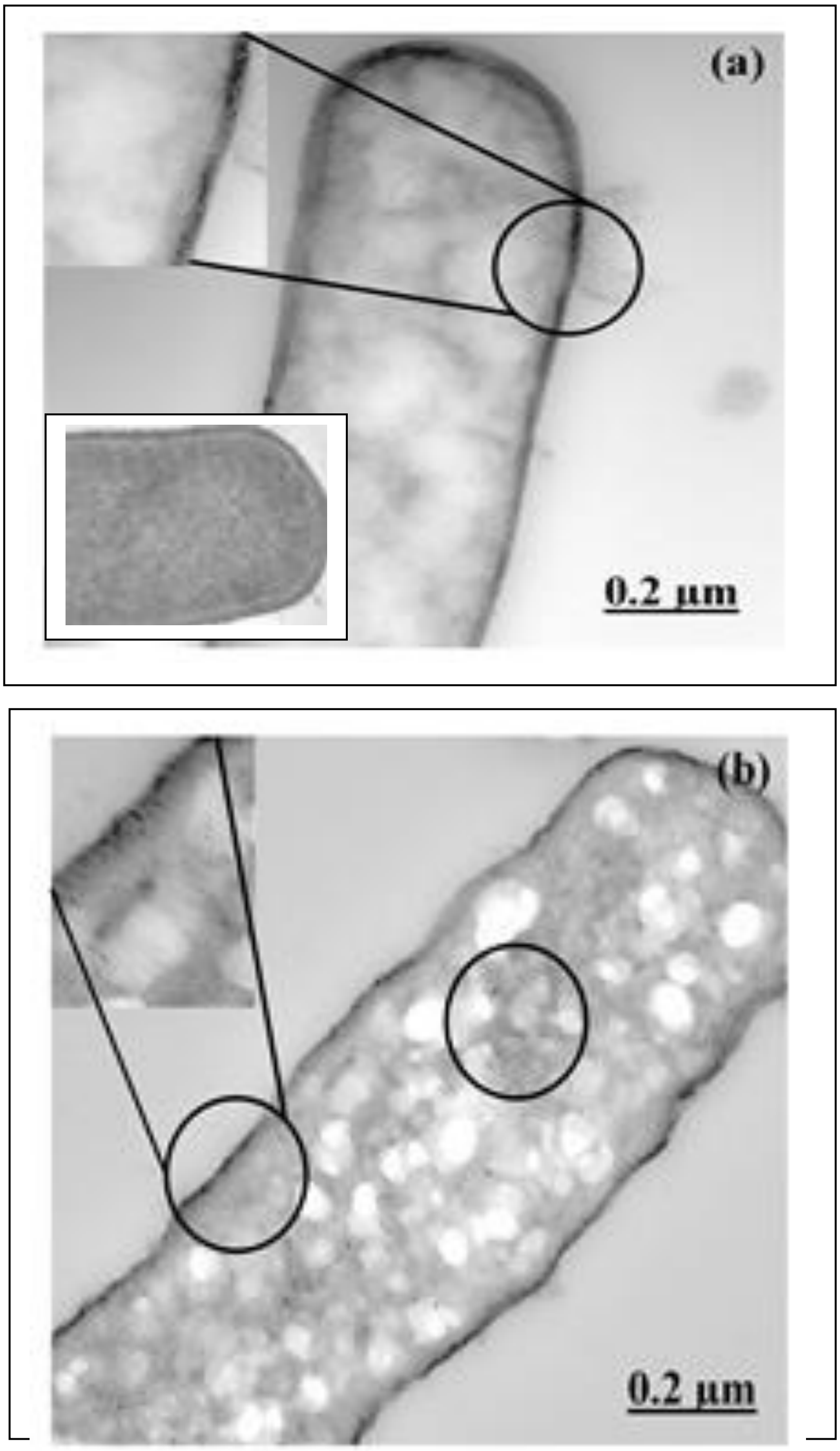

TEM images of cell sections of nominally (Table 1) 20wt% bio-Ru (a) and 20wt% bio-Pd/Ru (b) made by *B. benzeovorans.* Bottom inset in (a) is cells without added metal. Inset in (b) shows enlarged area of cell. Occasional NPs (circled) are visible only in cells that contained bio-Pd/Ru. Samples were viewed under a transmission electron microscope (JEOL 1200 EX; accelerating voltage of 80 kV[30]. Bars are 200 nm.

Figure S4

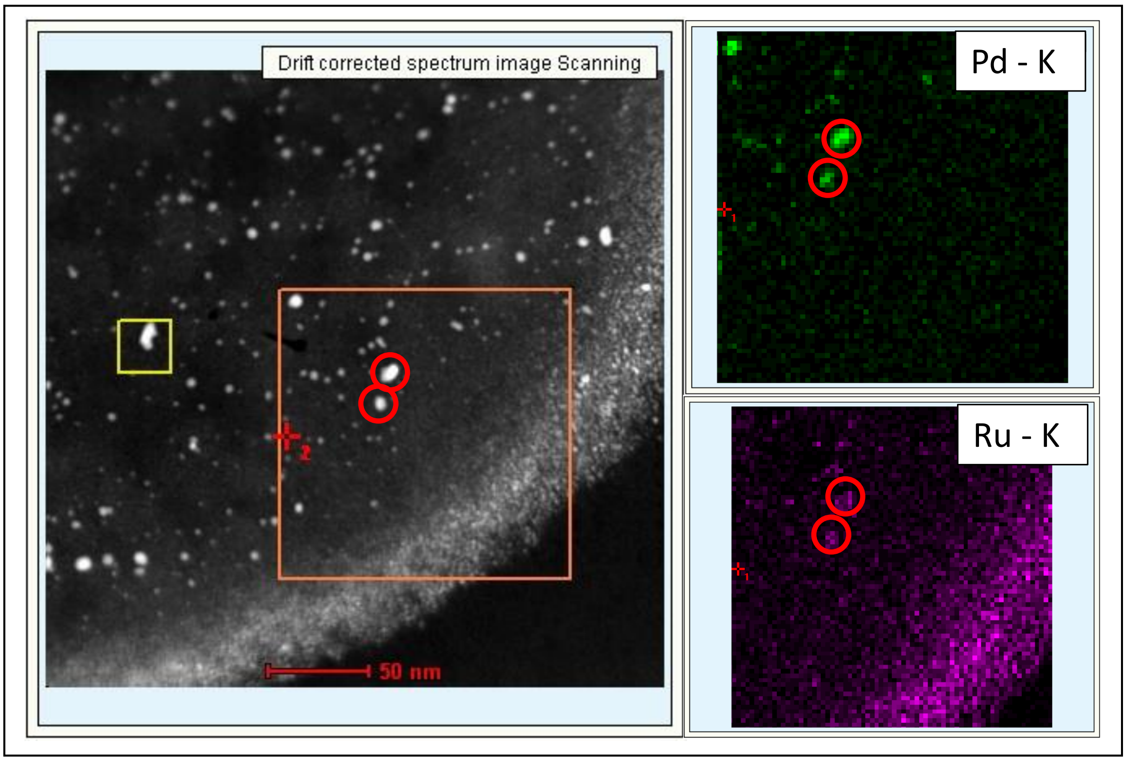

Co-localization of Pd and Ru in nanoparticles in the cytoplasm of *B. benzeovorans* by EDX-mapping. Note paucity of Pd at the cell wall (c.f. Fig. S2).

Figure S5 EDX analysis of bio-Pd/Ru on whole cells of *B. benzeovorans*

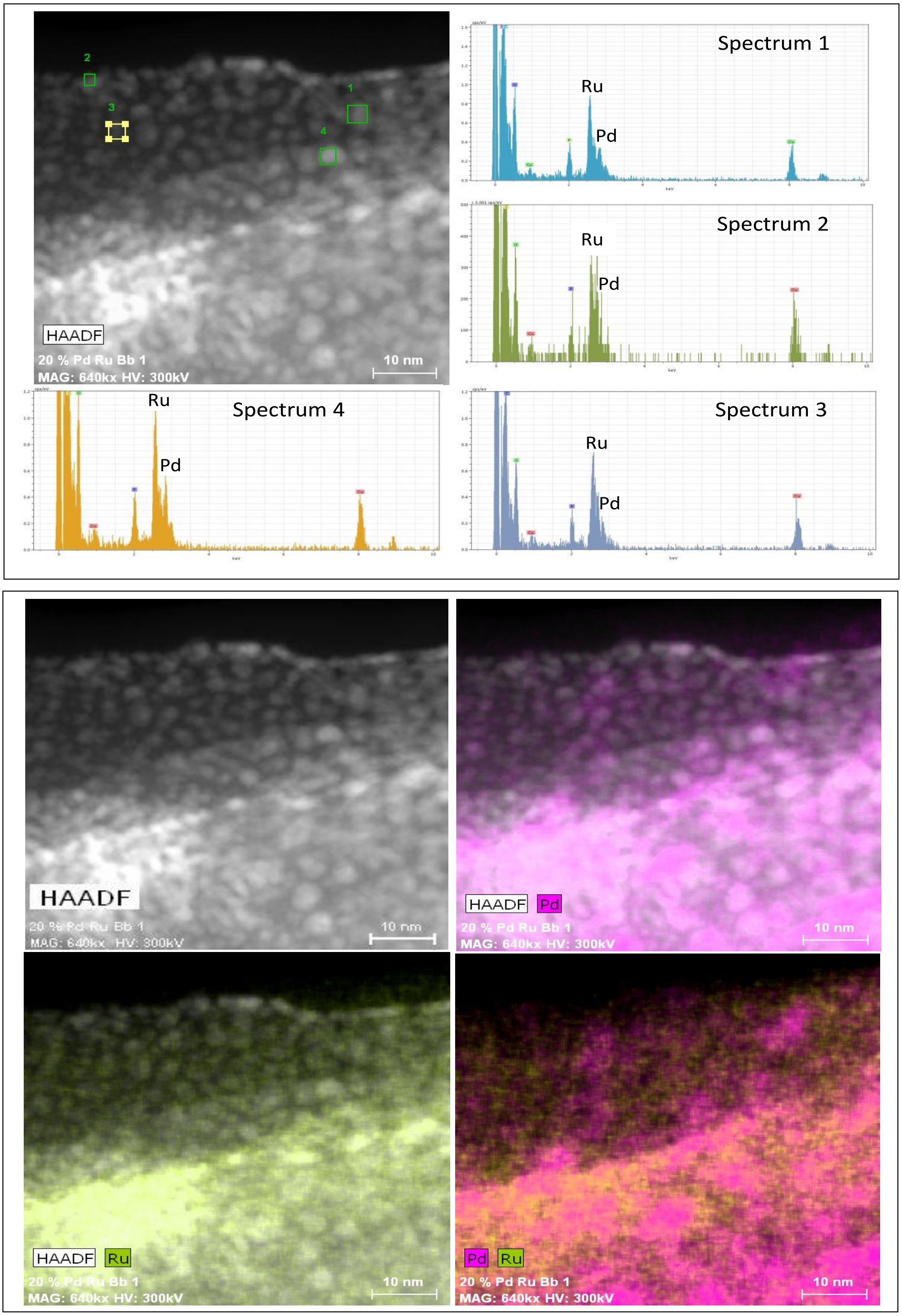

Figure S6

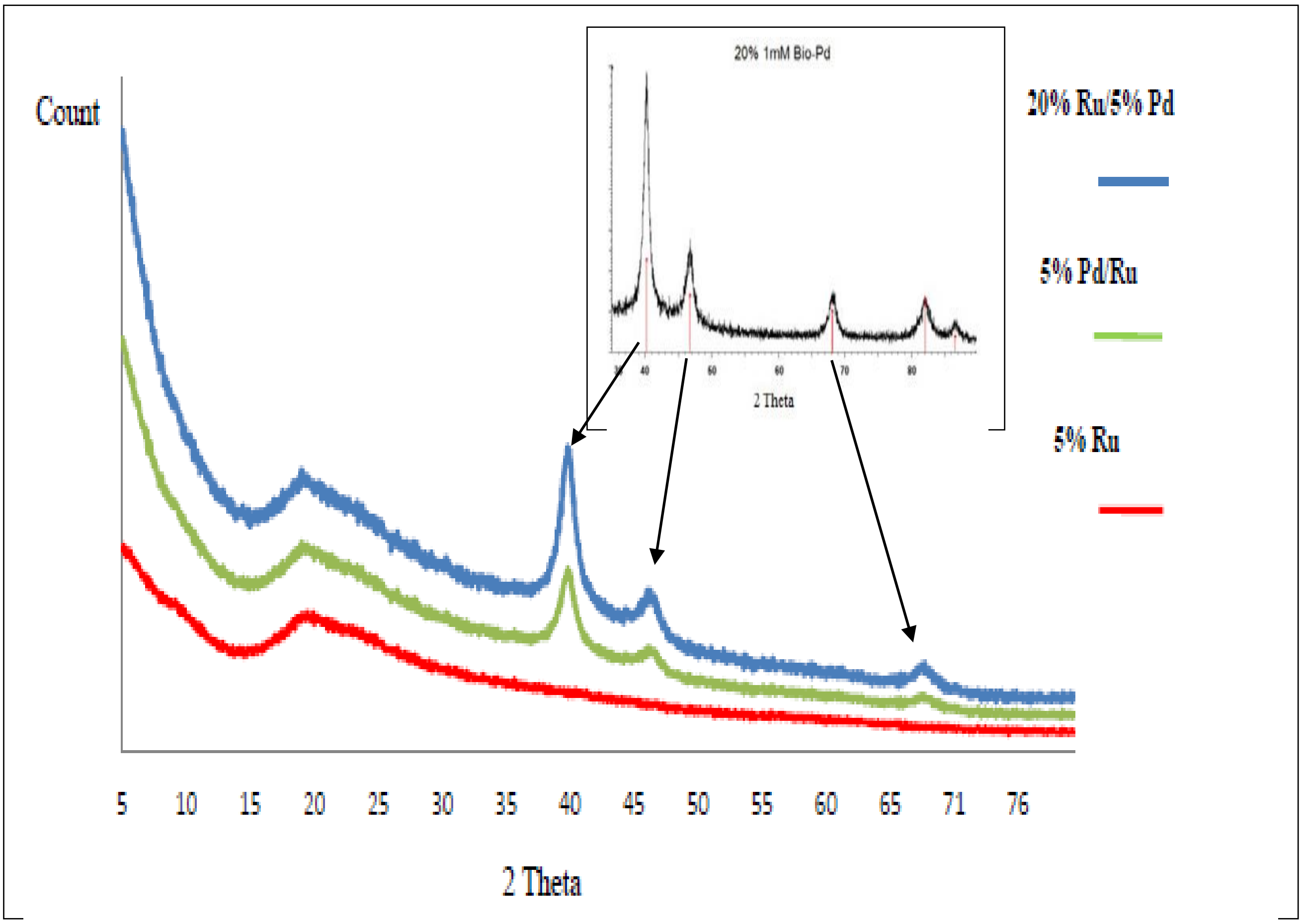

X-ray powder pattern of bio-Ru and bio Pd/Ru preparations together with 20 wt% bio-Pd (inset). Arrows correspond to the peaks at 2-theta° corresponding to 40.64° (Pd(111)), 46.15° (Pd(200)) and 68.27° (Pd(220)). The Pd peak positions and shape are unaffected by low or high dosing with Ru and hence there was no contraction of the Pd lattice by Ru and no evidence of alloying[37]; there is a large lattice mismatch between fcc Pd and hcp Ru[37].

Figure S7

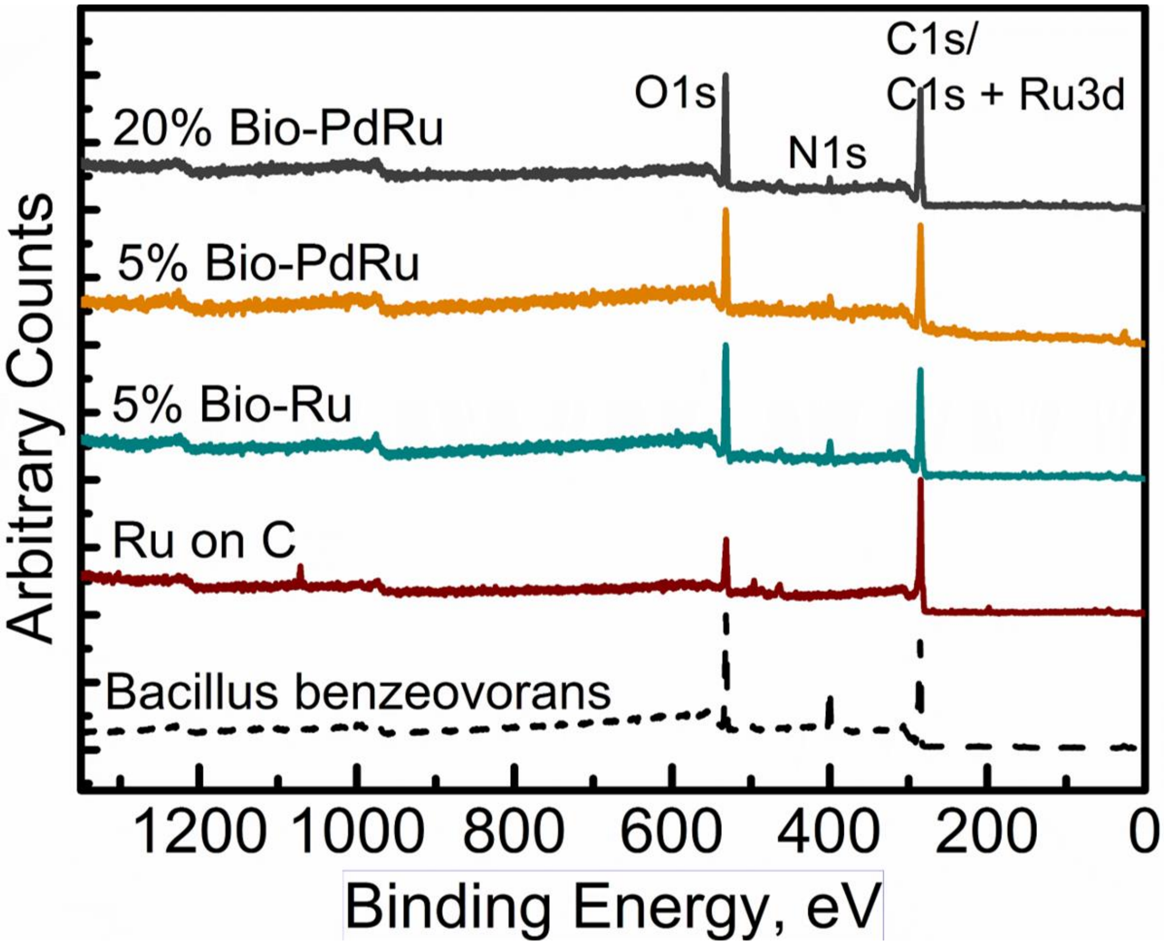

Wide energy XPS spectra for bio-Ru and bioPd/Ru bimetallic samples (nominal loadings are shown: c.f. Table 1) with, for comparison, spectra of *B. benzeovorans* (non-metallised cells) and Ru deposited on C (commercial sample).

Figure S8

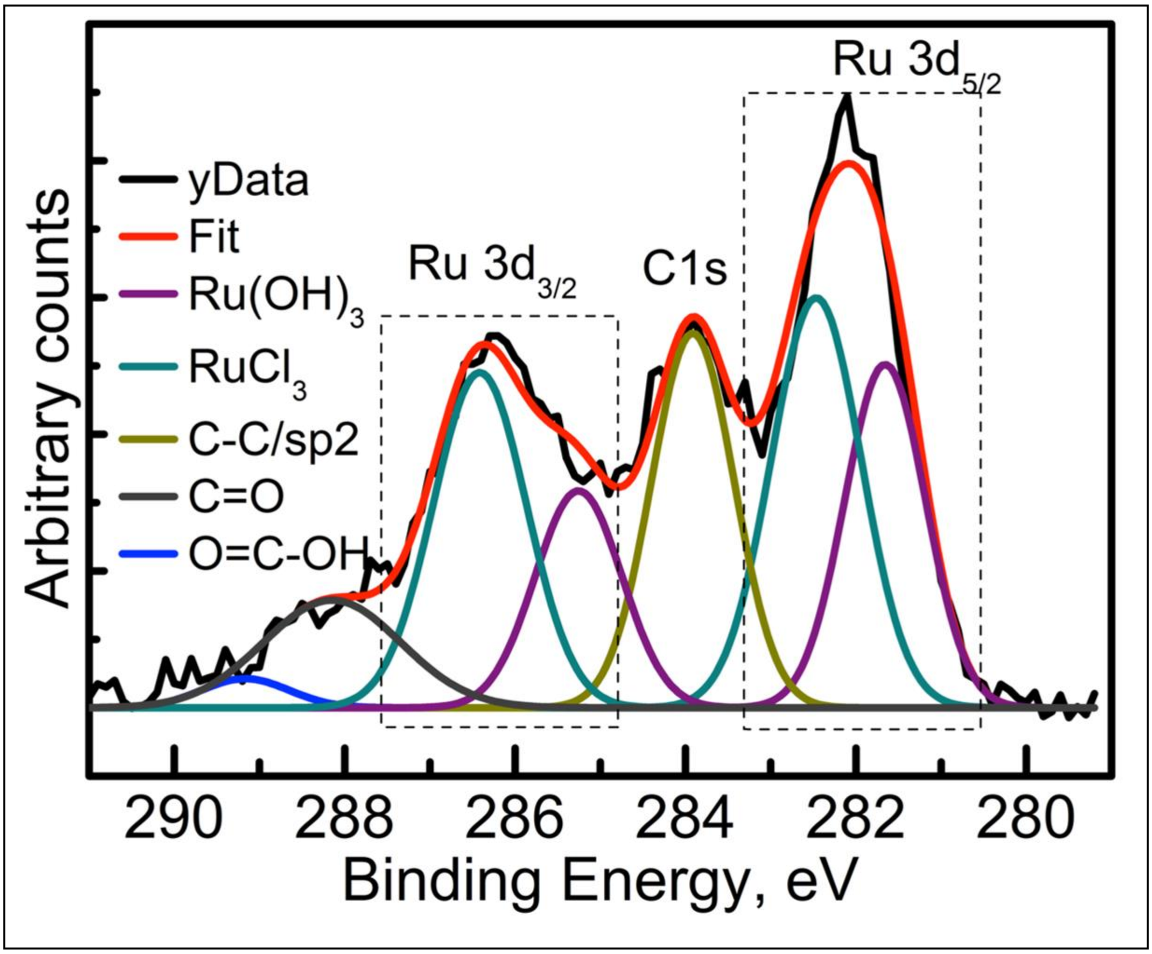

Spectra acquired for $RuCl_3$ powder with its resolved components as described in the main text.

Figure S9

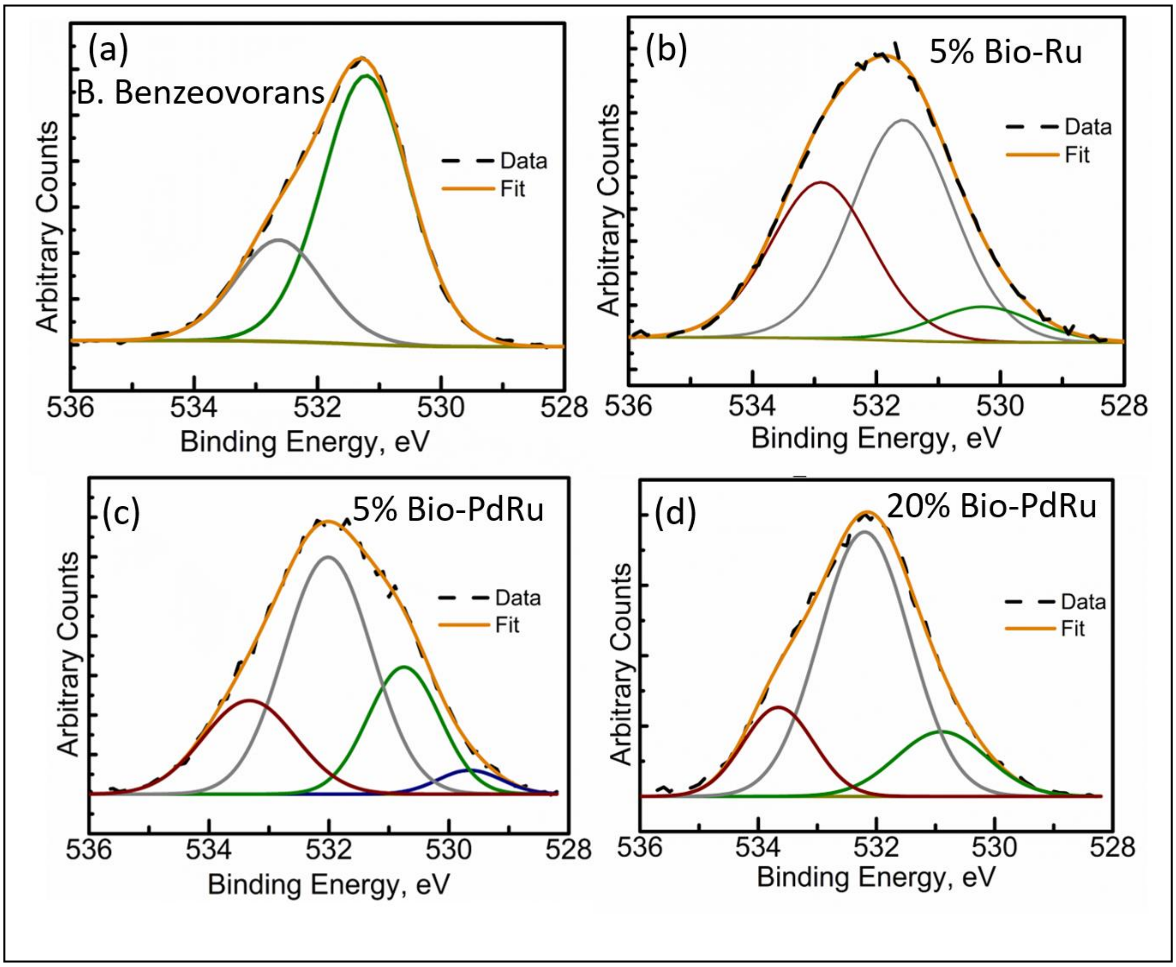

High resolution XPS O1s spectra for cells of *B. benzeovorans* (a), 5 wt% bio-Ru (b), 5 wt% bio-Pd/Ru (c) and 20wt% bio-Pd/Ru (d). Nominal metal loadings (see Table 1 for actual compositions)

Figure S10.

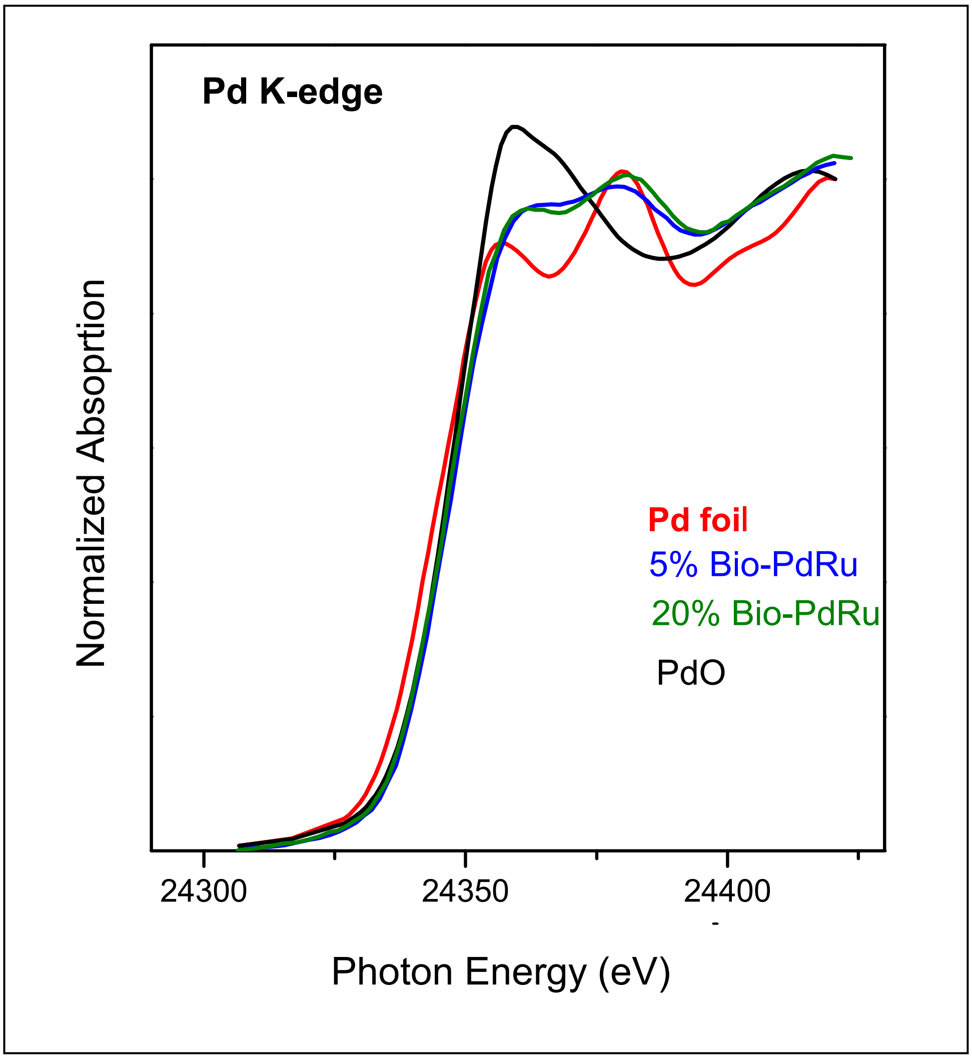

XANES region of EXAFS spectra of the Pd K-edge in reference compounds (Pd foil and PdO) and for biogenic Pd-Ru NPs samples

Figure S11

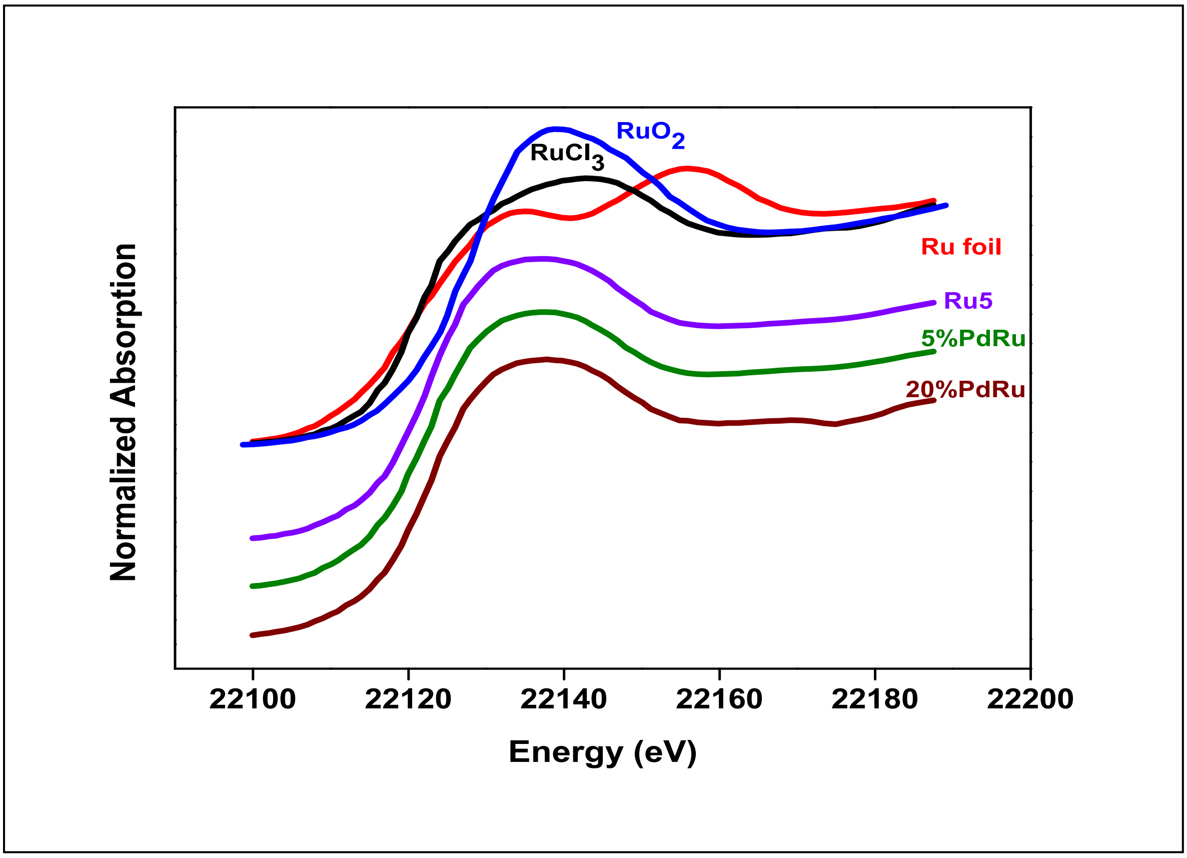

XANES spectra of ruthenium foil, RuO_2, $RuCl_3$, 5% Ru and of 5wt% and 20wt% Pd-Ru
